# Does COVID-19 change dietary habits and lifestyle behaviours in Kuwait: a community-based cross-sectional study

**DOI:** 10.1186/s12199-020-00901-5

**Published:** 2020-10-12

**Authors:** Wafaa Husain, Fatemah Ashkanani

**Affiliations:** Home Economics department, College of Basic Education, The Public Authority for Applied Education and Training, Al-Ardiya, Kuwait

**Keywords:** Dietary habits, Lifestyle, COVID-19, Kuwait

## Abstract

**Background:**

The coronavirus pandemic has transformed and continues to transform and affect the daily lives of communities worldwide, particularly due to the lockdown restrictions. Therefore, this study was designed to understand the changes in dietary and lifestyle behaviours that are major determinants of health during the COVID-19 outbreak.

**Methods:**

A cross-sectional study was conducted through an online questionnaire using a convenience sample of 415 adults living in Kuwait (age range 18–73 years).

**Results:**

The rate of skipping breakfast remained consistent, with a slight increase during the pandemic. Lunch remained the main reported meal before and during COVID-19. Compared to before COVID-19, people were much more likely have a late-night snack or meal during COVID-19 (OR = 3.57 (95% CI 1.79–7.26), *p* < 0.001). Moreover, there was a drastic decrease in the frequency of fast-food consumption during COVID-19, up to 82% reported not consuming fast food (*p* < 0.001). There was a significant increase in the percentage of participants who had their main meal freshly made (OR = 59.18 (95% CI 6.55–1400.76), *p* = 0.001). Regarding food group patterns, no significant differences were found before and during the pandemic in terms of the weekly frequency of consumption, except in the case of fish and seafood. There were no remarkable changes in beverage consumption habits among participants before and during the pandemic, except for Americano coffee and fresh juice. Furthermore, there was a great reduction in physical activity and an increase in the amount of screen time and sedentary behaviours. A notable increase was detected in day-time sleep and a decrease in night-time sleep among participants.

**Conclusion:**

In general, this study indicates some changes in daily life, including changes in some eating practices, physical activity and sleeping habits during the pandemic. It is important that the government considers the need for nutrition education programmes and campaigns, particularly during this critical period of the pandemic in Kuwait.

## Background

The World Health Organization (WHO) has declared the coronavirus disease 2019 (COVID-19) outbreak a pandemic, since it is spreading rapidly worldwide. It has affected more than 200 countries around the world [1]. It is a new disease that attacks the human respiratory system and can cause mild to severe illness [2]. Elderly and people with underlying medical conditions, such as cardiovascular diseases, hypertension, diabetes and cancer, are at a higher risk of death as a result of contracting COVID-19 [3–6]. In addition, it can cause severe complications among people with obesity-related conditions [7].

There is insufficient information concerning the risk factors that can lead to severe illness and there is no vaccine or specific treatment to prevent or cure the disease. Therefore, WHO recommendations have focused on avoiding contracting the virus through the practice of good hygiene, social distancing and only leaving the house when necessary [8]. On the 24th of February, the Kuwait Ministry of Health announced the first coronavirus cases. At the time of writing this report, in Kuwait, the number of confirmed COVID-19 cases had reached 75,697 and there had been more than 494 registered deaths [9].

In line with international action and in order to counter the spread of COVID-19, the government postponed study in all schools and universities and suspended work, a part from that of the emergency services. In addition, all malls and local businesses such as salons and gyms were closed [9]. Kuwait imposed a partial nationwide curfew on the 22nd of March 2020 until further notice. The government then imposed a total lockdown from the 10th to the 31st of May 2020. Furthermore, people were also encouraged to eat a healthy and balanced diet, be physically active and maintain a healthy lifestyle to support their immune system during these difficult times. The COVID-19 pandemic and government measures to stem its spread resulted in increased stress induced by the disruption of daily routine, along with fear and anxiety regarding the spread of the disease and its consequences for people’s finances, work, family and personal matters.

The relationship between stress and emotional eating is well established. Previous studies have shown an association between stress and the amount of food consumed [10–13]. It has been shown that people under stress crave more high fat and high sugar foods, since the body under stress requires more energy to function [14]. In addition, the body increases storage of abdominal fat [15].

It has been hypothesized that the increase in unstructured time and the psychological impact resulting from the enforced quarantine might induce changes in dietary habits and lifestyle. Therefore, the primary aim of this study was to investigate the effects of the COVID-19 outbreak during the lockdown on eating habits and other health-related behaviours among adults in Kuwait. Second is to examine the demographic variation in eating habits and lifestyle.

## Methods

### Design and data collection

A cross-sectional study was conducted in Kuwait during the COVID-19 outbreak from the 30th of March to the 15th of April 2020. Recruitment completely stopped before the holy month of Ramadan, which started on the 23rd of April, since it leads to marked changes in usual food patterns. A web-based survey tool (SurveyMonkey®) [16] was used among a convenience sample to investigate changes to their dietary habits and lifestyle during this period. Participants were recruited via online advertisements on social media through an invitation letter, with a link to the questionnaire and a request to circulate the survey broadly to adults (snowball sampling) in order to recruit more participants. Study information was provided at the start of the survey. It was anonymous and participation was voluntary. Respondents who were healthy and at least 18 years of age and above living in Kuwait could participate in the study. Respondents excluded from the study were those (1) less than 18 years of age; (2) infected with COVID-19; (3) who had illnesses/conditions that can affect their normal eating, including pregnancy; (4) not living in Kuwait; and (5) who did not complete the questionnaire appropriately. The study was conducted according to the guidelines laid down in the Declaration of Helsinki, and all procedures involving participants were conducted after obtaining agreement.

The minimum sample size required was calculated so that the sample proportion would be within 0.05 of the population proportion (which was assumed to be 0.50), with a 95% confidence level and a population size of 3,460,812 million [17]; thus, a minimum sample size of 385 was required.

A total of *n* = 510 questionnaires were completed. However, only 415 (81.4%) were valid. People who were less than 18 years of age (*n* = 43), pregnant (*n* = 7), not living in Kuwait (*n* = 25), or did not complete the questionnaire appropriately (*n* = 20) were excluded from the final analysis.

### Questionnaire

The questionnaire was developed by the primary investigators following a review of related literature [18–20]. It was evaluated and assessed by a number of experts, and specific modifications were made where needed. In addition, the questionnaire was piloted as a paper version among (*n* = 10) participants to check clarity and the suitability of wording, as well as the average time needed for its completion. The questionnaire was then amended to address all comments and converted into a web-based survey to increase response rates and reduce participant burden.

The questionnaire was divided into three parts. The first part used 10 questions to gather data about socio-demographic characteristics, including date of birth, sex, nationality, marital status, health status, education level and smoking habit. Moreover, the respondents were asked for their height, weight and their weight perception. The second part consisted of 30 questions and investigated dietary patterns and habits. Participants were asked about their usual meal patterns, food preparation, frequency of consumption of selected foods and food groups and frequency of consumption of selected beverages. The final part of the questionnaire asked about physical activity and lifestyle. It consisted of 6 questions aimed at investigating participants’ physical activity level and sleeping habits. Sleeping time (including naps) on a typical day was reported. All questions were asked in pairs in order to assess the same habits before and during the pandemic. For this study, ‘before the pandemic’ is defined as ‘the period before the COVID-19 outbreak’, and ‘during the pandemic’ is defined as ‘the period during the enforced partial quarantine in Kuwait’.

### Anthropometry

Height and weight information obtained in the questionnaire was all self-reported by the respondents. Body mass index (BMI) was then calculated as the ratio of weight in kilogrammes to height in metres squared. Weight status was classified according to WHO [21] categories as follows: underweight (BMI < 18.5), normal weight (BMI between 18.5–24.9), overweight (BMI between 25 and 29.9) and obese (BMI ≥ 30).

### Statistical analysis

Data was statistically analysed using Statistical Package for the Social Sciences, version 23 [22]. Descriptive statistics (means and standard deviations, or frequencies) were calculated for all variables. Variables were checked for normality by inspection and by using the Shapiro-Wilk. A chi-square (*χ*^2^) statistical test was conducted (univariate approach) to examine the significant differences for categorical variables. Multiple logistic regression analysis was used to investigate the possible changes of meal patterns, food group patterns, beverage consumption habits and physical activity between two periods of time (before and during the COVID-19 outbreak). A backward elimination (Wald) was applied. General linear model (GLM) repeated measures ANOVA was used to examine the demographic variation (age, BMI, gender, smoking habits, nationality, education level and marital status) in eating habits and lifestyle. All reported *p* values were 2-tailed, and *p* < 0.05 was considered to be statistically significant.

## Results

### Participants’ characteristics and weight status based on BMI category

The socio-demographic characteristics of the study participants are presented in Table 1. In total, 415 adults participated in this descriptive cross-sectional study with a mean age of 38.47 ± 12.73 years; most of them were females, numbering 285 (68.7%). The average BMI was 28.52 ± 6.741 kg/m^2^, which is indicative of the overweight category according to the definition of the WHO (male 30.36 ± 6.40 kg/m^2^, female 27.68 ± 6.73 kg/m^2^, *p* < 0.001). The majority of the sample was Kuwaiti. With regard to marital status, a majority of the sample were married, followed by single, divorced and widowed. In terms of education level, just over three quarters of the participants were highly educated.

**Table 1:** Socio-demographic characteristics of the study participants

Demographic characteristics of the study		All (***n*** = 415) ***n*** (%)
Age (years)	Mean ± SD (minimum–maximum)	38.47 ± 12.73 18–73
Gender	Male	130 (31.3)
	Female	285 (68.7)
BMI	Underweight	7 (1.7)
	Normal	116 (28)
	Overweight	154 (37.2)
	Obese	137 (33.1)
Nationality	Kuwaiti	376 (90.6)
	Non-Kuwaiti	39 (9.4)
Marital status	Single	132 (31.8)
	Married	235 (56.6)
	Divorced	43 (10.4)
	Widow	5 (1.2)
Education level	Less than high school	4 (1)
	High school	30 (7.2)
	Special courses	4 (1)
	Diploma	60 (14.5)
	Bachelor	221 (53.3)
	Postgraduate	96 (23.1)
Smoking habit	Yes	82 (19.8)
	No	333 (80.2)

*n* number, *SD* standard deviation

### Meal patterns before and during the pandemic

There was a drop in the reported number of times the majority of participants ate per day, from 4 times before the pandemic to 3 times during, although this was non-significant. Lunch remained the main reported meal before and during COVID-19. There was no significant change in the reported number of those skipping breakfast (*p* = 0.055), a meal snack (*p* = 0.255) and lunch (*p* = 0.830) between these two periods (*p* = 0.055). It was noticed that breakfast was picked as the most commonly skipped meal among participants during both periods. There was, however, significantly less skipping of the meal snack between lunch and dinner during COVID-19 than prior to COVID-19 (OR = 0.49 (95% CI 0.29–0.81), *p* = 0.006). In addition, compared to before COVID-19, people were much more likely to usually have a late-night snack or meal during COVID-19 (OR = 3.57 (95% CI 1.79–7.26), *p* < 0.001). The vast majority of participants, both before and during the pandemic, had their main meal freshly made. However, during the pandemic, there was a significant increase in the percentage of participants who had their main meal freshly made (OR = 59.18 (95% CI 6.55–1400.76), *p* = 0.001). Furthermore, the percentage of people who obtained their main meal from a restaurant also noticeably reduced, although this was not significant. Before the pandemic, 49% of the participants were more likely to consume fast food 1–2 times per week, while during COVID-19 up to 82% reported not consuming fast food (*p* < 0.001). There was no significant difference in terms of the person who prepared the food for the family before and during COVID-19. However, there was an increase in the percentage of participants who cooked for themselves, as well as wives who cooked during COVID-19, accompanied by a reduction in the percentage of participants who relied on a housekeeper for cooking during COVID-19. With regard to stress eating, it was noticed that the percentage of participants who described themselves as usually engaging in stress eating increased, although no significant differences were detected between both periods (Table 2). Only age and educational level had a significant effect among participants (see Supplementary Table A, Additional File 1).

**Table 2:** Multivariate analysis between two periods using logistic regression for meal pattern

Meal pattern	Multiple-choice responses	Before ***n*** (%)	During ***n*** (%)	OR (95% CI)	OR (95% CI)
How many times a day do you eat?	One time	5 (1.2)	4 (1.0)	-	-
	Two times	56 (13.5)	43 (10.4)	0.96 (0.24–4.08)	0.29 (0.05–1.68)
	Three times	124 (29.9)	112 (27.0)	1.13 (0.29–4.66)	0.36 (0.06–1.99)
	Four times	131 (31.6)	104 (25.1)	0.99 (0.26–4.10)	0.36 (0.06–2.02)
	Five times	80 (19.3)	89 (21.4)	1.39 (0.36–5.79)	0.43 (0.08–2.47)
	Six or more	19 (4.6)	63 (15.2)	4.14 (1.00–18.26)	0.88 (0.14–5.70)
What meal would you consider to be your main meal?	Dinner	49 (11.8)	102 (24.6)	1.10 (0.44–2.60)	1.56 (0.44–5.25)
	Breakfast	78 (18.8)	58 (14.0)	0.39 (0.16–0.93)	0.42 (0.12–1.41)
	Lunch	279 (67.2)	238 (57.3)	0.45 (0.19–1.01)	0.66 (0.20–2.07)
	Other	9 (2.2)	17 (4.1)	-	-
Skipping meal breakfast	No	254 (61.2)	242 (58.3)	-	-
	Yes	161 (38.8)	173 (41.7)	1.13 (0.85–1.49)	0.62 (0.38–1.01)
Skipping meal snack (breakfast and lunch)	No	281 (67.7)	308 (74.2)	-	-
	Yes	134 (32.3)	107 (25.8)	0.73 (0.54–0.98)	0.77 (0.49–1.21)
Skipping meal lunch	No	378 (91.1)	358 (86.3)	-	-
	Yes	37 (8.9)	57 (13.7)	1.63 (1.05–2.54)	0.93 (0.48–1.81)
Skipping meal snack between lunch and dinner	No	295 (71.1)	363 (87.5)	-	-
	Yes	120 (28.9)	52 (12.5)	0.35 (0.24–0.50)	0.49 (0.29–0.81)
Skipping meal dinner	No	296 (71.3)	331 (79.8)	-	-
	Yes	119 (28.7)	84 (20.2)	0.63 (0.46–0.87)	0.64 (0.38–1.08)
None skipping meal	No	400 (96.4)	392 (94.5)	-	-
	Yes	15 (3.6)	23 (5.5)	1.56 (0.81–3.10, *p* = 0.187)	0.58 (0.22–1.53)
How likely are you to have a late night snack or meal? (past 10 pm)	Never	89 (21.4)	65 (15.7)	-	-
	Rarely	135 (32.5)	82 (19.8)	0.83 (0.55–1.27)	0.77 (0.45–1.32)
	Occasionally	140 (33.7)	136 (32.8)	1.33 (0.90–1.98)	1.65 (0.96–2.87)
	Usually	51 (12.3)	132 (31.8)	3.54 (2.26–5.62)	3.57 (1.79–7.26)
How is your main meal prepared?	Freshly made	306 (73.7)	386 (93.0)	13.88 (2.68–254.35)	59.18 (6.55–1400.76)
	Restaurant	61 (14.7)	9 (2.2)	1.62 (0.26–31.45)	11.91 (1.11–305.06)
	Microwave	37 (8.9)	19 (4.6)	5.65 (0.98–107.13)	22.56 (2.29–556.04)
	None	116 (28.0)	341 (82.2)	-	-
Number of times a week you consume fast food	Other	11 (2.7)	1 (0.2)	-	-
	1–2/week	205 (49.4)	57 (13.7)	0.09 (0.07–0.13)	0.09 (0.06–0.14)
	3–4/week	72 (17.3)	9 (2.2)	0.04 (0.02–0.08)	0.04 (0.02–0.09)
	5 or more/week	22 (5.3)	8 (1.9)	0.12 (0.05–0.27)	0.10 (0.03–0.28)
Who prepares and cooks in your family	By my self	86 (20.7)	133 (32.0)	1.55 (0.58–4.11)	1.43 (0.37–5.50)
	Husband	1 (0.2)	2 (0.5)	2.00 (0.16–47.71)	6.96 (0.29–279.42)
	Wife	59 (14.2)	70 (16.9)	1.19 (0.44–3.23)	0.96 (0.25–3.72)
	Father	0 (0.0)	1 (0.2)	-	-
	Mother	82 (19.8)	74 (17.8)	0.90 (0.34–2.43)	1.05 (0.27–4.11)
	Grandparents	9 (2.2)	6 (1.4)	0.67 (0.16–2.65)	1.06 (0.15–6.98)
	Housekeeper	169 (40.7)	120 (28.9)	0.71 (0.27–1.87)	0.64 (0.17–2.40)
	Other	9 (2.2)	9 (2.2)	-	-
Do you eat when you feel stressed, unhappy, angry, or bored?	Never	100 (24.1)	98 (23.6)	-	-
	Rarely	103 (24.8)	87 (21.0)	0.86 (0.58–1.28)	0.96 (0.57–1.62)
	Occasionally	149 (35.9)	143 (34.5)	0.98 (0.68–1.41)	1.12 (0.69–1.83)
	Usually	63 (15.2)	87 (21.0)	1.41 (0.92–2.17)	1.18 (0.62–2.28)

*n* number, *OR* odds ratio, *CI* confidence interval

### Food group patterns

There was no significant difference among participants in terms of the weekly frequency of consumption of red meat, chicken, processed meat, canned fish, fruits, vegetables, bread, milk, cooking fat and most snack foods, both before and during the pandemic.

Nearly half of the participants, both before and during COVID-19, consumed red meat 1–2 times per week (49.4% and 47.5% respectively).

With regard to chicken consumption, nearly half of the participants, both before and during COVID-19, consumed chicken 3–4 times per week (41.4% and 39.5% respectively).

There was an overall significant reduction in the frequency of consumption of fish and seafood. More participants preferred to consume fish and seafood the most, once to twice weekly compared to never, both before and during COVID-19 (OR = 0.25, 95% CI 0.15–0.40, *p* < 0.001). Furthermore, there was a great increase in the percentage of participants who reported that they did not eat any fish or seafood (from 10.6 to 26.5%).

More than half of the participants never consumed canned fish (52.8%, 58.1%), and the majority never consumed processed meat (68.4%, 69.4%) before and during. With regard to fruit consumption, the most commonly reported frequency was 1 serving per day both before and during. In terms of vegetable consumption, the most commonly reported frequency was 1 serving per day both before and during. It can be seen from the data in Table 3 that most participants did not meet the USDA minimum recommended daily intake of fruits and vegetables of 5 servings a day (2 servings of fruits and 3 servings of vegetables). A total of 76.9% and 73.8% of participants did not meet the fruit consumption recommendation both before and during respectively. Moreover, about 86% of participants did not meet the vegetable consumption recommendation both before and during the pandemic.

**Table 3:** Multivariate analysis between two periods using logistic regression for food groups pattern

Food groups pattern	Multiple-choice responses	Before ***n*** (%)	During ***n*** (%)	OR (95% CI)	OR (95% CI)
Red meat	Never	32 (7.7)	42 (10.1)	-	-
	Less than 1/w	72 (17.3)	86 (20.7)	0.91 (0.52–1.58)	1.00 (0.51–1.95)
	1–2/w	205 (49.4)	197 (47.5)	0.73 (0.44–1.20)	0.97 (0.52–1.81)
	3–4/w	94 (22.7)	75 (18.1)	0.61 (0.35–1.05)	0.80 (0.40–1.59)
	5–6/w	5 (1.2)	9 (2.2)	1.37 (0.43–4.83)	1.27 (0.33–5.24)
	7 or more	3 (0.7)	2 (0.5)	0.51 (0.06–3.24)	0.82 (0.09–6.48)
	I don’t know	4 (1.0)	4 (1.0)	0.76 (0.17–3.44)	1.27 (0.21–7.79)
Chicken	Never	15 (3.6)	20 (4.8)	-	-
	Less than 1/w	18 (4.3)	27 (6.5)	1.12 (0.46–2.77)	1.85 (0.64–5.40)
	1–2/w	149 (35.9)	143 (34.5)	0.72 (0.35–1.45)	1.35 (0.55–3.26)
	3–4/w	172 (41.4)	164 (39.5)	0.72 (0.35–1.44)	1.24 (0.51–2.99)
	5–6/w	44 (10.6)	47 (11.3)	0.80 (0.36–1.75)	1.14 (0.43–2.99)
	7 or more	13 (3.1)	11 (2.7)	0.63 (0.22–1.80)	0.72 (0.20–2.55)
	I don’t know	4 (1.0)	3 (0.7)	0.56 (0.10–2.92)	0.48 (0.06–3.77)
Fish and sea food	Never	44 (10.6)	110 (26.5)	-	-
	Less than 1/w	131 (31.6)	137 (33.0)	0.42 (0.27–0.64)	0.36 (0.22–0.58)
	1–2/w	195 (47.0)	143 (34.5)	0.29 (0.19–0.44)	0.25 (0.15–0.40)
	3–4/w	36 (8.7)	18 (4.3)	0.20 (0.10–0.38)	0.15 (0.07–0.31)
	5–6/w	1 (0.2)	1 (0.2)	0.40 (0.02–10.26)	0.23 (0.01–9.79)
	7 or more	2 (0.5)	1 (0.2)	0.20 (0.01–2.14)	0.17 (0.01–2.14)
	I don’t know	6 (1.4)	5 (1.2)	0.33 (0.09–1.16)	0.27 (0.05–1.34)
Processed meat	Never	284 (68.4)	288 (69.4)	-	-
	Less than 1/w	74 (17.8)	67 (16.1)	0.89 (0.62–1.29)	0.92 (0.61–1.40)
	1–2/w	38 (9.2)	29 (7.0)	0.75 (0.45–1.25)	0.75 (0.41–1.33)
	3–4/w	13 (3.1)	21 (5.1)	1.59 (0.79–3.32)	1.82 (0.81–4.18)
	5–6/w	2 (0.5)	4 (1.0)	1.97 (0.38–14.31)	1.10 (0.18–9.19)
	7 or more	0 (0.0)	1 (0.2)	-	-
	I don’t know	4 (1.0)	5 (1.2)	1.23 (0.32–5.02)	1.56 (0.25–11.85)
Canned fish	Never	219 (52.8)	241 (58.1)	-	-
	Less than 1/w	132 (31.8)	99 (23.9)	0.68 (0.49–0.94)	0.79 (0.55–1.13)
	1–2/w	46 (11.1)	48 (11.6)	0.95 (0.61–1.48)	1.26 (0.77–2.07)
	3–4/w	10 (2.4)	15 (3.6)	1.36 (0.61–3.19)	1.50 (0.61–3.77)
	5–6/w	1 (0.2)	3 (0.7)	2.73 (0.35–55.33)	1.11 (0.06–50.08)
	7 or more	0 (0.0)	1 (0.2)	-	-
	I do not know	7 (1.7)	8 (1.9)	1.04 (0.37–3.01)	0.98 (0.27–3.53)
Fruit	None	33 (8.0)	38 (9.2)	-	-
	Less than 1/d	129 (31.1)	121 (29.2)	0.81 (0.48–1.38)	0.76 (0.41–1.41)
	1/d	157 (37.8)	147 (35.4)	0.81 (0.48–1.36)	0.82 (0.44–1.55)
	2/d	62 (14.9)	76 (18.3)	1.06 (0.60–1.89)	1.16 (0.57–2.35)
	3/d	17 (4.1)	18 (4.3)	0.92 (0.41–2.08)	1.29 (0.49–3.38)
	4 or more	9 (2.2)	7 (1.7)	0.68 (0.22–2.01)	0.71 (0.19–2.51)
	I do not know	8 (1.9)	8 (1.9)	0.87 (0.29–2.61)	1.06 (0.25–4.45)
Vegetables	None	29 (7.0)	34 (8.2)	-	-
	Less than 1/d	93 (22.4)	96 (23.1)	0.88 (0.50–1.56)	1.09 (0.57–2.09)
	1/d	151 (36.4)	140 (33.7)	0.79 (0.46–1.36)	0.97 (0.51–1.85)
	2/d	85 (20.5)	89 (21.4)	0.89 (0.50–1.59)	0.92 (0.46–1.82)
	3/d	32 (7.7)	38 (9.2)	1.01 (0.51–2.01)	1.02 (0.45–2.31)
	4 or more	17 (4.1)	9 (2.2)	0.45 (0.17–1.14)	0.46 (0.15–1.34)
	I do not know	8 (1.9)	9 (2.2)	0.96 (0.33–2.86)	1.02 (0.27–3.90)
Bread group	Other	10 (2.4)	7 (1.7)	-	-
	White	178 (42.9)	199 (48.0)	1.60 (0.60–4.48)	1.44 (0.46–4.72)
	Brown/brown seeds	183 (44.1)	164 (39.5)	1.28 (0.48–3.60)	1.13 (0.37–3.65)
	Whole wheat	39 (9.4)	40 (9.6)	1.47 (0.51–4.40)	1.55 (0.47–5.31)
	Seeds	0 (0.0)	0 (0.0)	-	-
	None	5 (1.2)	5 (1.2)	1.43 (0.29–7.12)	2.33 (0.39–14.37)
Milk group	None	97 (23.4)	99 (23.9)	-	-
	Whole milk	124 (29.9)	128 (30.8)	1.01 (0.70–1.47)	1.15 (0.76–1.76)
	Semi-skimmed	99 (23.9)	103 (24.8)	1.02 (0.69–1.51)	1.14 (0.73–1.78)
	Skimmed	56 (13.5)	47 (11.3)	0.82 (0.51–1.33)	1.09 (0.64–1.87)
	Soy milk	4 (1.0)	5 (1.2)	1.22 (0.32–5.08)	1.93 (0.45–8.71)
	Almond milk	21 (5.1)	15 (3.6)	0.70 (0.34–1.43)	0.94 (0.40–2.18)
	Other (rice/goat milk)	11 (2.7)	13 (3.1)	1.16 (0.49–2.76)	1.07 (0.41–2.84)
	Do not know	3 (0.7)	5 (1.2)	1.63 (0.39–8.14)	2.60 (0.37–23.68)
Fat type	None	19 (4.6)	13 (3.1)	-	-
	Butter	21 (5.1)	19 (4.6)	1.32 (0.52–3.43)	1.14 (0.39–3.40)
	Vegetable oil	234 (56.4)	241 (58.1)	1.51 (0.73–3.19)	1.58 (0.69–3.75)
	Olive oil	109 (26.3)	107 (25.8)	1.43 (0.68–3.11)	1.49 (0.64–3.61)
	Ghee/lard	7 (1.7)	8 (1.9)	1.67 (0.49–5.90)	1.52 (0.40–5.95)
	Others	9 (2.2)	10 (2.4)	1.62 (0.52–5.20)	1.59 (0.45–5.77)
	Do not know	16 (3.9)	17 (4.1)	1.55 (0.58–4.20)	1.86 (0.59–6.00)
**Type of snack**					
Biscuit	No	346 (83.4)	320 (77.1)	-	-
	Yes	69 (16.6)	95 (22.9)	1.49 (1.06–2.11)	1.86 (1.26–2.75)
Crisps	No	289 (69.6)	284 (68.4)	-	-
	Yes	126 (30.4)	131 (31.6)	1.06 (0.79–1.42)	0.90 (0.63–1.27)
Chocolate	No	236 (56.9)	229 (55.2)	-	-
	Yes	179 (43.1)	186 (44.8)	1.07 (0.81–1.41)	1.12 (0.83–1.53)
Nuts	No	283 (68.2)	276 (66.5)	-	-
	Yes	132 (31.8)	139 (33.5)	1.08 (0.81–1.44)	1.05 (0.75–1.45)
Vegetables and fruits	No	300 (72.3)	284 (68.4)	-	-
	Yes	115 (27.7)	131 (31.6)	1.20 (0.89–1.62)	1.28 (0.90–1.82)

*n* number, *OR* odds ratio, *CI* confidence interval, *w* week, *d* day

Brown/brown seeded bread was the most frequently consumed type of bread before COVID-19 (44.1%), followed closely by white bread (42.9%). On the other hand, white bread was the most frequently consumed during (48%), followed by brown/brown seeded bread (39.5%). With regard to the type of milk consumed, whole milk was the most frequently consumed milk (29.9%, 30.8%), followed by semi-skimmed milk (23.9%, 24.8%), before and during respectively. Furthermore, vegetable oil was the most frequently used fat for cooking (56.4%, 58.1%), followed by olive oil (26.3%, 25.8%), before and during.

With regard to favourite snacks, the most commonly reported snacks by the participants were chocolate followed by nuts and crisps before and during respectively. There was a significant between-subject effect of gender on food group patterns among participants (*p* < 0.01), but not for BMI, age, marital status, nationality, smoking status and educational level (see Supplementary Table B, Additional File 1).

### Beverage consumption habits

There was no significant difference in the number of beverages consumed per day among the participants for Arabic coffee, tea, fizzy drinks, energy drinks, fruit drinks, herbal tea and water, both before and during the pandemic. However, most participants consumed Americano coffee 1–2 times per day compared to none per day, both before and during COVID-19 (OR = 0.5, 95% CI 0.40–0.85, *p* = 0.005). Likewise, participants were less likely to drink fresh juice 1–2 times per day compared to none per day, both before and during COVID-19 (OR = 0.51, 95% CI 0.32–0.83, *p* = 0.006) (Table 4). BMI and smoking status were noted to be the only parameters to have a significant between-subject effect among participants (*p* = 0.027 and *p* = 0.001 respectively) (see Supplementary Table C, Additional File 1).

**Table 4:** Multivariate analysis between two periods using logistic regression for beverage consumption habits

Beverages	Multiple-choice responses	Before ***n*** (%)	During ***n*** (%)	OR (95% CI)	OR (95% CI)
Americano coffee	None	94 (22.7)	131 (31.6)	-	-
	Less than 1	100 (24.1)	106 (25.5)	0.76 (0.52–1.11)	0.81 (0.54–1.21)
	1–2/d	172 (41.4)	137 (33.0)	0.57 (0.40–0.81)	0.58 (0.40–0.85)
	3–4/d	36 (8.7)	23 (5.5)	0.46 (0.25–0.82)	0.53 (0.28–0.99)
	5–6/d	8 (1.9)	12 (2.9)	1.08 (0.43–2.84)	2.19 (0.76–6.69)
	More than 6/d	5 (1.2)	6 (1.4)	0.86 (0.25–3.07)	0.71 (0.17–2.88)
Arabic coffee	None	192 (46.3)	227 (54.7)	-	-
	Less than 1	72 (17.3)	57 (13.7)	0.67 (0.45–0.99)	0.73 (0.48–1.12)
	1–2/d	64 (15.4)	56 (13.5)	0.74 (0.49–1.11)	0.74 (0.47–1.15)
	3–4/d	44 (10.6)	32 (7.7)	0.62 (0.37–1.01)	0.62 (0.36–1.06)
	5–6/d	24 (5.8)	23 (5.5)	0.81 (0.44–1.49)	0.90 (0.47–1.72)
	More than 6/d	19 (4.6)	20 (4.8)	0.89 (0.46–1.73)	0.83 (0.40–1.70)
Tea	None	117 (28.2)	114 (27.5)	-	-
	Less than 1	112 (27.0)	103 (24.8)	0.94 (0.65–1.37)	1.08 (0.72–1.62)
	1–2/d	131 (31.6)	137 (33.0)	1.07 (0.75–1.53)	1.34 (0.91–1.98)
	3–4/d	44 (10.6)	41 (9.9)	0.96 (0.58–1.57)	1.14 (0.66–1.97)
	5–6/d	7 (1.7)	12 (2.9)	1.76 (0.68–4.88)	2.34 (0.83–6.99)
	More than 6/d	4 (1.0)	8 (1.9)	2.05 (0.63–7.86)	2.11 (0.55–9.19)
Fizzy drinks	None	263 (63.4)	267 (64.3)	-	-
	Less than 1	91 (21.9)	89 (21.4)	0.96 (0.69–1.35)	1.02 (0.70–1.49)
	1–2/d	49 (11.8)	44 (10.6)	0.88 (0.57–1.37)	1.05 (0.63–1.74)
	3–4/d	8 (1.9)	9 (2.2)	1.11 (0.42–2.99)	1.36 (0.44–4.24)
	5–6/d	3 (0.7)	4 (1.0)	1.31 (0.29–6.72)	1.11 (0.23–6.05)
	More than 6/d	1 (0.2)	2 (0.5)	1.97 (0.19–42.54)	2.31 (0.20–52.98)
Energy drinks	None	370 (89.2)	382 (92.0)	-	-
	Less than 1	27 (6.5)	21 (5.1)	0.75 (0.41–1.35)	0.61 (0.31–1.17)
	1–2/d	16 (3.9)	11 (2.7)	0.67 (0.30–1.44)	0.45 (0.17–1.13)
	3–4/d	2 (0.5)	1 (0.2)	0.48 (0.02–5.08)	0.43 (0.02–4.93)
	5–6/d	0 (0.0)	0 (0.0)	-	-
	More than 6/d	0 (0.0)	0 (0.0)	-	-
Fruit drinks	None	246 (59.3)	250 (60.2)	-	-
	Less than 1	106 (25.5)	102 (24.6)	0.95 (0.68–1.31)	1.11 (0.77–1.61)
	1–2/d	61 (14.7)	57 (13.7)	0.92 (0.61–1.37)	1.11 (0.70–1.77)
	3–4/d	1 (0.2)	5 (1.2)	4.92 (0.79–94.62)	7.30 (0.93–158.59)
	5–6/d	0 (0.0)	0 (0.0)	-	-
	More than 6/d	1 (0.2)	1 (0.2)	0.98 (0.04–24.98)	4.06 (0.14–121.29)
Fresh juice	None	227 (54.7)	258 (62.2)	-	-
	Less than 1	122 (29.4)	107 (25.8)	0.77 (0.56–1.06)	0.68 (0.48–0.96)
	1–2/d	64 (15.4)	44 (10.6)	0.60 (0.39–0.92)	0.51 (0.32–0.83)
	3–4/d	2 (0.5)	6 (1.4)	2.64 (0.60–18.14)	1.93 (0.36–15.82)
	5–6/d	0 (0.0)	0 (0.0)	-	-
	More than 6/d	0 (0.0)	0 (0.0)	-	-
Herbal tea	None	215 (51.8)	228 (54.9)	-	-
	Less than 1	115 (27.7)	101 (24.3)	0.83 (0.60–1.15, *p* = 0.257)	0.88 (0.61–1.25)
	1–2/d	76 (18.3)	78 (18.8)	0.97 (0.67–1.40, *p* = 0.861)	1.04 (0.70–1.55)
	3–4/d	9 (2.2)	5 (1.2)	0.52 (0.16–1.54, *p* = 0.253)	0.57 (0.17–1.74)
	5–6/d	0 (0.0)	3 (0.7)	1997406.56 (0.00–NA, *p* = 0.977)	1251915.00 (0.00–NA)
	More than 6/d	0 (0.0)	0 (0.0)	-	-
Water	None	22 (5.3)	15 (3.6)	-	-
	Less than 1	83 (20.0)	79 (19.0)	1.40 (0.68–2.93, *p* = 0.367)	1.56 (0.72–3.46)
	1–2/d	133 (32.0)	149 (35.9)	1.64 (0.82–3.36, *p* = 0.162)	1.75 (0.83–3.81)
	3–4/d	101 (24.3)	88 (21.2)	1.28 (0.63–2.66, *p* = 0.502)	1.38 (0.64–3.07)
	5–6/d	76 (18.3)	84 (20.2)	1.62 (0.79–3.40, *p* = 0.192)	1.84 (0.84–4.13)
	More than 6/d	0 (0.0)	0 (0.0)	-	-

*n* number, *OR* odds ratio, *CI* confidence interval, *d* day

### Physical activity and sleeping habits

With regard to practising a physical activity, before the pandemic, 18% of the participants exercised only in some seasons, compared to 20% who never did. During COVID-19, only 10% of participants reported exercising only in some seasons, compared to 39.5% who reported not exercising at all (OR = 0.36, 95% CI 0.21–0.62, *p* < 0.001). There was, however, no significant difference in the number of hours of physical activity per week among the participants, both before and during the pandemic. Before COVID-19, 14% of participants spent their free time shopping compared to walking (87%). However, during COVID-19, this percentage significantly dropped to 2.2% compared to walking (11.6%) (OR = 0.19, 95% CI 0.07–0.45, *p* < 0.001). The percentage of participants who spent more than 6 h per day watching TV or on a computer/phone increased from 16.1% before COVID-19 to 43.6% during COVID-19 (OR = 3.84, 95% CI 2.33–6.42, *p* < 0.001), compared to spending just 1–2 h per day on these devices (Table 5).

**Table 5:** Multivariate analysis between two periods using logistic regression for physical activity

Physical activity	Multiple-choice responses	Before ***n*** (%)	During ***n*** (%)	OR (95% CI)	OR (95% CI)
Practising a physical activity	Never	84 (20.2)	164 (39.5)	-	-
	Only in some seasons	78 (18.8)	43 (10.4)	0.28 (0.18–0.44)	0.36 (0.21–0.62)
	Sometimes	167 (40.2)	147 (35.4)	0.45 (0.32–0.63)	0.74 (0.46–1.19)
	Always	86 (20.7)	61 (14.7)	0.36 (0.24–0.55)	1.09 (0.53–2.27)
Hours practising PA per week	Less than 1 h or none	203 (48.9)	257 (61.9)	-	-
	1–2 h/w	84 (20.2)	75 (18.1)	0.71 (0.49–1.01)	0.86 (0.52–1.41)
	3–4 h/w	61 (14.7)	49 (11.8)	0.63 (0.42–0.96)	0.88 (0.48–1.61)
	More than 4 h/w	67 (16.1)	34 (8.2)	0.40 (0.25–0.63)	0.57 (0.26–1.24)
Free time activity spend	Walking	87 (21.0)	48 (11.6)	-	-
	TV, music, computer, reading	230 (55.4)	339 (81.7)	2.67 (1.82–3.97)	1.40 (0.87–2.28)
	Sports	37 (8.9)	19 (4.6)	0.93 (0.48–1.78)	0.73 (0.34–1.57)
	Shopping	61 (14.7)	9 (2.2)	0.27 (0.12–0.56)	0.19 (0.07–0.45)
Hours spend on computer/mobile/TV	1–2 h/d	126 (30.4)	50 (12.0)	-	-
	3–4 h/d	138 (33.3)	79 (19.0)	1.44 (0.94–2.22)	1.32 (0.81–2.15)
	5–6 h/d	84 (20.2)	105 (25.3)	3.15 (2.05–4.90)	2.30 (1.39–3.82)
	More than 6 h/d	67 (16.1)	181 (43.6)	6.81 (4.45–10.56)	3.84 (2.33–6.42)
Sleeping time habit	Night sleep	268 (66.3)	119 (29.7)	-	-
	Day sleep	136 (33.7)	282 (70.3)	4.67 (3.48–6.31)	3.99 (2.86–5.62)
Sleeping amount	Mean (SD)	7.1 (1.7)	8.0 (2.1)	1.29 (1.20–1.40)	1.28 (1.17–1.40)

*n* number, *OR* odds ratio, *CI* confidence interval, *h* hour, *w* week, *d* day, *SD* standard deviation

With regard to sleeping habits, results indicated significant statistical differences before and during the pandemic, there was a decrease in the percentage of participants who slept during the night and a marked increase in the percentage of participants who slept during the day (OR = 3.99 (95% CI 2.86–6.62), *p* < 0.001).

There was a significant between-subject effect of BMI (*p* = 0.001) and age (*p* = 0.003) on physical activity, but not for smoking status, gender, marital status, nationality or educational level (see Supplementary Table D, Additional File 1).

## Discussion

This study produces novel information about dietary habits and lifestyle behaviours in Kuwait during the period of COVID-19. This critical period resulted in not only serious public health consequences, but also severe economic and social consequences globally [23]. Maintaining a healthy and balanced diet and being physically active are recommended in these difficult times to support the immune system [1–3]. However, factors such as sudden lifestyle changes, anxiety, fear, stress and depression can influence food choices and everyday behaviours [24]. The present findings seem to be consistent with other research that has observed changes in dietary habits [25–27] and lifestyle behaviours [28] during the pandemic. The findings of this study indicate some changes in daily life, including changes in some eating practices, physical activity and sleeping habits. Unhealthy meal patterns were detected in this study, such as skipping breakfast and late-night snacking. Both behaviours are likely associated with overweight and obesity [29, 30]. Consistent with the literature [31–34], this research found skipping breakfast was common among participants. It was noticed that the rate of skipping breakfast remained consistent, with a slight increase during the pandemic. Possible explanations for this behaviour include a lack of time, intentionally skipping breakfast to cut calories and a lack of appetite [33]. However, other possible explanations for skipping breakfast during COVID-19 include staying up late, which leads to late-night snacking, and oversleeping during the day, as shown in the results. These findings reflect those of Okada et al. [30], which was a study among 19,687 Japanese women that found a significant association between a late dinner or bedtime snack and skipping breakfast, as well as an association of this behaviour with overweight and obesity.

Despite the recommendation to reduce the intake of fats, sugar and salt during COVID-19 [35] and avoid irregular snacking [36], chocolate, nuts and crisps were reported to be the most commonly consumed snacks, and these are loaded with sugar, fat and salt. A similar finding was reported by Scarmozzino and Visioli [25], who found that half of the participants of an Italian sample showed an increase in the consumption of both sweet and salty comfort foods during COVID-19. These results may be explained by the fact that feelings of boredom, anxiety and stress (likely heightened due to quarantine, as shown in the results) lead to higher consumption of energy-dense foods that are high in sugar and fat [14, 37, 38]. Similar findings were also reported by Muscogiuri et al. [39], who found that many people over-eat sugary and salty comfort foods for snacking due to stress induced by quarantine and that this habit may increase the risk of developing obesity. Furthermore, it has been demonstrated that there was a strong association between weight gain and self-reported anxiety/depression among patients during the pandemic [26].

The results of this study indicate a rise in home cooking during COVID-19. Participants started cooking more themselves (or their wives and mothers did so), resulting in reduced reliance on a housekeeper for cooking purposes. These results match those observed by an American study [40] that found about half of the participants reported they were cooking and baking more during the pandemic. Moreover, this result matches with the findings of Di Renzo [41] and Scarmozzino and Visioli [25] that there was an increased consumption of homemade foods such as desserts, bread and pizza during the lockdown among Italian residents.

Furthermore, the study detected a significant reduction in the frequency of fast-food consumption. It seems possible that this rise in home cooking is related to attempts to occupy the increased free time resulting from quarantine. Another explanation is that people wanted to eat healthier in reaction to the spread of COVID-19 and thus resorted to home cooking more frequently. Finally, it could be related to the reduced consumption of fast-food as a result of fears regarding the transmission of COVID-19, whether it be from unhygienic practices at restaurants or from the delivery driver. However, it is difficult to conclude that people ate more healthily during the pandemic just because they reported consuming more home cooked meals, especially if unhealthy foods were still in circulation.

Regarding food choices within the five main food groups assessed in this study, there were no significant changes in terms of red meat, chicken, type of fat, milk, bread, fruit and vegetables, before and during the pandemic, except in the case of fish and seafood. A study reported by Zhao et al. [27] also observed low consumptions of fish during the lockdown among the Chinese population. This result was expected and can be explained by the fact that the fish markets in Kuwait were closed on April 2020 [9] as a precautionary measure and so there was a lack of availability of fresh fish and seafood due to the absence of working fisherman during this period. In addition, it is often preferred to consume fish and seafood fresh, which may have further contributed to the reduction in their consumption.

Although the consumption of fruits and vegetables is recommended to support the immune system especially during the pandemic [35], however, the results of this study show that more than 70% of the participants did not reach the minimum portions of fruits and vegetables recommended by the USDA of 5 portions a day [42]. This result is in line with findings from other studies that reported a low consumption of fruits and vegetables among Kuwaiti adults [43] such as the EMAN study [44] and KNNS [45]. These results are likely related to a lack of awareness of the current recommendation for the consumption of fruits and vegetables (unpublished data). A finding from two cross-sectional questionnaire studies among the UK’s population found an association between low knowledge of details of the 5-a-day recommendation and low consumption of fruits and vegetables [46]. In addition, another possible explanation is a predisposition towards energy-dense foods that are high in sugar and fat for snacking, as shown in the results. Moreover, the limited availability of fruits and vegetables and restricted food store opening hours due to quarantine during the pandemic could have caused a reduction in the consumption of fruits and vegetables.

The results of this study did not show remarkable changes in beverage consumption habits among participants before and during the pandemic, except for Americano coffee and fresh juice, which both showed a reduction in consumption during COVID-19. Regarding Americano coffee, a possible explanation for the reduction in its consumption might be the closing of coffee shops as a precautionary measure during the pandemic. A study conducted in Kuwait that gives support to this explanation is Allafi et al. [47], which found that most of its participants preferred to drink their coffee at coffee shops and that the most consumed type of coffee daily was Americano. The reduction in the consumption of fresh juice can possibly be explained by the negative impact of the pandemic on the availability of fruit and vegetables, since Kuwait has very limited agricultural production, particularly in terms of fruit. Moreover, fresh fruit and vegetables have short shelf lives. In addition, making fresh juice requires a larger amount of fruit and vegetables than when simply eating them. Another possible explanation is the limited access to grocery shopping, as elaborated on above.

It has been suggested that to enhance the immune system, it is important to be physically active and get enough sleep [48]. In the present study, a noticeable reduction was found in the prevalence of physical activity during COVID-19, while time spent on sedentary behaviours increased, similar to the findings of Ammar et al. [28]. This is most likely due to social distancing measures and the need for open spaces for people to be physically active [49].

Based on the results, more than half of the participants met the recommended sleeping hours during the pandemic. However, 70% of the participants slept during the daytime instead of night-time. This result may be explained by the fact that quarantine may cause stress, which results in sleep disturbances and abnormal sleep patterns, or because of changes in daily routine. This may negatively affect the immune system [50]. Moreover, it may increase food intake and increase the risk of developing obesity [39].

Various challenges and difficulties were faced during the recruitment stage of the study. There was a lack of interest in the study accompanied by a lack of motivation to take part despite possible interest. This was particularly true during the early stage of the pandemic, likely due to the panic and stress induced by this unfamiliar situation resulting in people not giving priority to participation in the study and instead focusing on preparation for the pandemic in terms of stockpiling food and other necessities in the limited time available to do so because of the enforced partial quarantine and the measures put in place to organize access to daily grocery shopping. The lack of motivation to take part may have been further exacerbated by the length of the questionnaire. Furthermore, there was a lack of time on the side of the researchers to recruit for the study, since they were bound by the approach of the holy month of Ramadan, which was due to start on the 23rd of April, and it causes marked changes in usual food patterns.

### Limitations of the study

It is acknowledged that the current study has some limitations. Firstly, all measurements, including height and weight, physical activity, dietary, smoking and sleeping habits, were self-reported. The poor informative status may increase information bias. Secondly, diet was only measured using questions that relied on daily or weekly frequency consumption; measuring of serving size was neglected. Moreover, the consumption substances that are specific dietary risk factors, such as fat, sodium and sugar, were not collected. Thirdly, although the study questionnaire was developed after a comprehensive review of literature, the tool was new, and this could add to the limitations. In addition, a web-based survey tool was used for its convenience during COVID-19 which may have led to selection bias. Most participants were highly educated. Furthermore, as a convenience sample was used in this study, the number of individuals who agreed to take part in the study could be one of the limitations.

## Conclusion

The present study provided, for the first time, information on the eating habits and lifestyle changes during the pandemic after the beginning of the lockdown period in Kuwait. The results are still preliminary and need to be confirmed with further studies among a larger population that also includes people with confirmed COVID-19. The poor dietary habits together with an unhealthy lifestyle can cause serious health problems. It is therefore important that the government considers the need for organized campaigns, workshops and nutrition education programmes to teach the foundations of nutrition, meal planning and how to adapt and maintain healthy eating and living practices. Extra consideration should be given to vulnerable groups such as pregnant women, children and elderly people. This is particularly important during this critical period of the pandemic in Kuwait. Furthermore, social media should be used more frequently as a means to step up nutrition advice by increasing the presence of experts in the field of nutrition such as dietitian, nutritionist, doctors and health workers. This need is more pressing during this period of absence of formal education.

## Supplementary information

**Additional file 1.** Supplementary tables.

## Data Availability

The datasets of this article are available from the corresponding author upon reasonable request.

## Abbreviations

WHO: World Health Organization
COVID-19: Coronavirus disease 2019
BMI: Body mass index
KNNS: Kuwait Nutrition Surveillance System
EMAN: Eastern Mediterranean Approach of non-communicable disease
FAO: Food and Agriculture Organization
