# Supplementary material for: Does COVID-19 change dietary habits and lifestyle behaviours in Kuwait: a community-based cross-sectional study
Source: Environ Health Prev Med. 2020 Oct 12;25:61. doi: 10.1186/s12199-020-00901-5 (PMC7548533; doi:10.1186/s12199-020-00901-5)
Supplement: Supplementary file 1 — Additional file 1. Supplementary tables. [file 12199_2020_901_MOESM1_ESM.docx]

**Table A Repeated measure for meal patterns to measure the influence from demographics and its between subjects’ effects**

|  | | **Sum of Squares** | | **df** | | **Mean Square** | | **F** | | **p** | | **η² _p_** | |
| --- | --- | --- | --- | --- | --- | --- | --- | --- | --- | --- | --- | --- | --- |
| BMI_categories |  | 13.916 |  | 3 |  | 4.639 |  | 2.344 |  | 0.073 |  | 0.017 |  |
| Age |  | 36.897 |  | 1 |  | 36.897 |  | 18.644 |  | < .001 |  | 0.044 |  |
| DoYouSmoke |  | 0.234 |  | 1 |  | 0.234 |  | 0.118 |  | 0.731 |  | 0.000 |  |
| Gender |  | 1.413 |  | 1 |  | 1.413 |  | 0.714 |  | 0.399 |  | 0.002 |  |
| MaritalStatus |  | 3.765 |  | 1 |  | 3.765 |  | 1.903 |  | 0.169 |  | 0.005 |  |
| Nationality |  | 0.338 |  | 1 |  | 0.338 |  | 0.171 |  | 0.680 |  | 0.000 |  |
| EducationLevel |  | 14.620 |  | 1 |  | 14.620 |  | 7.388 |  | 0.007 |  | 0.018 |  |
| Residual |  | 799.509 |  | 404 |  | 1.979 |  |  |  |  |  |  |  |
|  | | | | | | | | | | | | | |
| *Note.*  Type III Sum of Squares | | | | | | | | | | | | | |

**Table B Repeated measure for Food groups pattern to measure the influence from demographics and its between subjects’ effect**

| **Cases** | | **Sum of Squares** | | **df** | | **Mean Square** | | **F** | | **p** | | **η²** | |
| --- | --- | --- | --- | --- | --- | --- | --- | --- | --- | --- | --- | --- | --- |
| BMI_categories |  | 9.815 |  | 3 |  | 3.272 |  | 0.808 |  | 0.490 |  | 4.755e -4 |  |
| Age |  | 15.734 |  | 1 |  | 15.734 |  | 3.886 |  | 0.049 |  | 7.622e -4 |  |
| Gender |  | 66.329 |  | 1 |  | 66.329 |  | 16.384 |  | < .001 |  | 0.003 |  |
| MaritalStatus |  | 2.833 |  | 1 |  | 2.833 |  | 0.700 |  | 0.403 |  | 1.373e -4 |  |
| Nationality |  | 6.324 |  | 1 |  | 6.324 |  | 1.562 |  | 0.212 |  | 3.064e -4 |  |
| EducationLevel |  | 13.277 |  | 1 |  | 13.277 |  | 3.280 |  | 0.071 |  | 6.432e -4 |  |
| DoYouSmoke |  | 4.273 |  | 1 |  | 4.273 |  | 1.056 |  | 0.305 |  | 2.070e -4 |  |
| Residuals |  | 1635.545 |  | 404 |  | 4.048 |  |  |  |  |  |  |  |
|  | | | | | | | | | | | | | |
| *Note.*  Type III Sum of Squares | | | | | | | | | | | | | |

**Table C Repeated measure for beverage consumption habits to measure the influence from demographics and its between subjects’ effect**

| **Cases** | **Sum of Squares** | **df** | **Mean Square** | **F** | **p** | **η²** |
| --- | --- | --- | --- | --- | --- | --- |
| Age | 3.281 | 1 | 3.281 | 1.388 | 0.239 | 4.045e -4 |
| Gender | 7.966 | 1 | 7.966 | 3.369 | 0.067 | 0.001 |
| MaritalStatus | 1.292 | 1 | 1.292 | 0.546 | 0.46 | 1.731e -4 |
| Nationality | 0.557 | 1 | 0.557 | 0.235 | 0.628 | 6.761e -5 |
| EducationLevel | 1.048 | 1 | 1.048 | 0.443 | 0.506 | 1.779e -4 |
| DoYouSmoke | 25.655 | 1 | 25.655 | 10.85 | 0.001 | 0.004 |
| BMI_categories | 11.692 | 1 | 11.692 | 4.944 | 0.027 | 0.002 |
| Residuals | 960.028 | 406 | 2.365 |  |  |  |
|  | | | | | | |
| *Note.*  Type III Sum of Squares | | | | | |  |

**Table D Repeated measure for Physical activity to measure the influence from demographics and its between subjects’ effect**

| **Cases** | | **Sum of Squares** | | **df** | | **Mean Square** | | **F** | | **p** | | **η²** | |
| --- | --- | --- | --- | --- | --- | --- | --- | --- | --- | --- | --- | --- | --- |
| BMI_categories |  | 23.384 |  | 3 |  | 7.795 |  | 3.817 |  | 0.010 |  | 0.004 |  |
| Age |  | 18.370 |  | 1 |  | 18.370 |  | 8.996 |  | 0.003 |  | 0.003 |  |
| Gender |  | 0.372 |  | 1 |  | 0.372 |  | 0.182 |  | 0.670 |  | 5.805e -5 |  |
| MaritalStatus |  | 1.617 |  | 1 |  | 1.617 |  | 0.792 |  | 0.374 |  | 2.527e -4 |  |
| Nationality |  | 0.041 |  | 1 |  | 0.041 |  | 0.020 |  | 0.888 |  | 6.333e -6 |  |
| EducationLevel |  | 0.877 |  | 1 |  | 0.877 |  | 0.429 |  | 0.513 |  | 1.370e -4 |  |
| DoYouSmoke |  | 5.542 |  | 1 |  | 5.542 |  | 2.714 |  | 0.100 |  | 8.660e -4 |  |
| Residuals |  | 794.347 |  | 389 |  | 2.042 |  |  |  |  |  |  |  |
|  | | | | | | | | | | | | | |
| *Note.*  Type III Sum of Squares | | | | | | | | | | | | | |
